# Prognostic Signatures of Alternative Splicing Events in Esophageal Carcinoma Based on TCGA Splice-Seq Data

**DOI:** 10.3389/fonc.2021.658262

**Published:** 2021-10-05

**Authors:** Ping Ye, Yan Yang, Liqiang Zhang, Guixi Zheng

**Affiliations:** ^1^ National Health Commission Key Laboratory of Otorhinolaryngology, Department of Otorhinolaryngology, Qilu Hospital of Shandong University, Jinan, China; ^2^ Department of Clinical Laboratory, Qilu Hospital of Shandong University, Jinan, China

**Keywords:** esophageal carcinoma, alternative splicing, splicing factor, TCGA, prognosis

## Abstract

An alternative splicing (AS) event is a highly complex process that plays an essential role in post-transcriptional gene expression. Several studies have suggested that abnormal AS events were the primary element in the pathological process of cancer. However, few works are dedicated to the study of AS events in esophageal carcinoma (EC). In the present study, clinical information and RNA-seq data of EC patients were downloaded from The Cancer Genome Atlas (TCGA) database. The percent spliced in (PSI) values of AS events were acquired from the TCGA Splice-seq. A total of 183 EC patients were enrolled in this study, and 2,212 AS events were found significantly associated with the overall survival of these patients by univariate Cox regression analysis. The prognostic signatures based on AS events were built by multivariate Cox analysis. Receiver operating characteristic (ROC) curves displayed that the area under the curve (AUC) of the following prognostic signatures, including exon skip (ES), alternate terminator (AT), alternate acceptor site (AA), alternate promoter (AP), alternate donor site (AD), retained intron (RI), and total events, was greater than 0.8, suggesting that these seven signatures had valuable prognosis prediction capacity. Finally, the risk score of prognostic signatures was indicated as an independent risk factor of survival. Gene Ontology (GO) and Kyoto Encyclopedia of Genes and Genomes (KEGG) pathway analyses were performed to explore the function of splicing factors (SFs) that were associated with AS events. Also, the interactive network between AS events and SFs identified several hub genes and AS events which need further study. This was a comprehensive study that explored prognosis-related AS events and established valuable prognosis signatures in EC patients. The network of interactions between AS events and SFs might offer novel insights into the fundamental mechanisms of tumorigenesis and progression of EC.

## Introduction

Esophageal carcinoma (EC) is the seventh most frequent cancer and the sixth leading cause of cancer mortality in the world according to the 2018 Global Cancer Statistics (1). There are 5.48 deaths and 5.90 new cases per 100,000 people worldwide annually (2). Surgery is still the first choice for the treatment of EC, regardless of whether auxiliary treatment is performed, which depends on the individual tumor stage (3). However, a large proportion of EC patients are diagnosed at a late stage because of vague symptoms, thus missing the opportunity to undergo early surgical curative treatment. More than 50% of newly diagnosed patients have irreversible lesions and distant metastases. The 5-year survival rate of EC patients is less than 20% (1, 2, 4). Although many studies have explored the potential carcinogenesis of EC, its underlying molecular mechanisms have not yet been clearly elucidated.

The alternative splicing (AS) event is a highly complex process that plays an essential role in post-transcriptional gene expression and is one of the foundations of biodiversity and complexity. Its regulation is multilayered and includes the inherent role of RNA structural arrangement, which will undergo temporal and tissue-specific changes (5). Previous studies have suggested that abnormal AS events were crucial in the pathological process of several diseases, including cancer. In the past decade, several studies have demonstrated the potential role of AS events in tumorigenesis and progression of cancer. In particular, advances in RNA sequencing technology and analysis have led to an increased AS functional relevance in cancer in recent years (6). The prognostic value of AS events has been demonstrated in several cancer types, such as breast cancer (7–9), hepatocellular carcinoma (10, 11), gastric cancer (12, 13), and others (14–23). However, there are few works dedicated to the study of AS events in EC.

In this study, 183 EC patients were enrolled and univariate Cox regression analysis determined that 2,212 AS events were meaningfully associated with the overall survival (OS) of these patients. Lasso and multivariate Cox regression analyses were employed to build prognostic signatures, and the prognostic value of each prognostic signature was demonstrated by Kaplan–Meier (K-M) and receiver operating characteristic (ROC) curves. Moreover, we identified splicing factors (SFs) associated with AS events and performed functional enrichment analyses. The regulatory relationship between AS events and SFs was also analyzed.

## Materials and Methods

### Data Compilation Process

Clinical and transcriptome data of the EC patients were acquired from The Cancer Genome Atlas (TCGA) database. The percent spliced in (PSI) values for AS events were downloaded from the TCGA Splice-seq, which is a collection of alternative mRNA splicing patterns in cancer (24).

### Survival Analysis

Univariate/multivariate Cox regression analyses were executed between the OS and AS events of the patients. Volcano and UpSet plots were carried out to demonstrate the survival-associated AS events. The PSI value was used as the basis for calculating the risk score, which was used to distribute patients into low- and high-risk groups. The K-M curve was applied to compare the survival time between these two groups. The ROC curve was employed and the area under the curve (AUC) was calculated to indicate the prognostic value of each signature. The survival time, risk score distribution, and expression heatmap of survival-related AS events were then visualized. Independent prognostic analysis was performed on the risk score and clinical information, including gender; clinical stage; and T, M, and N stages.

### Functional Enrichment Analysis

Gene Ontology (GO) functional enrichment and Kyoto Encyclopedia of Genes and Genomes (KEGG) pathway enrichment analyses of the SFs were performed. GO analysis results included molecular function (MF), cell composition (CC), and biological process (BP). Terms with *P*-value <0.05 were considered significant categories in both GO and KEGG.

### AS–SF Regulation Network

The correlation between survival-associated AS events and SF genes was analyzed by Pearson *t*-test. Then, the network of interactions between AS events and SFs was constructed and visualized by the Cytoscape software.

## Results

### Clinical Features and AS Events

**Figure 1:**
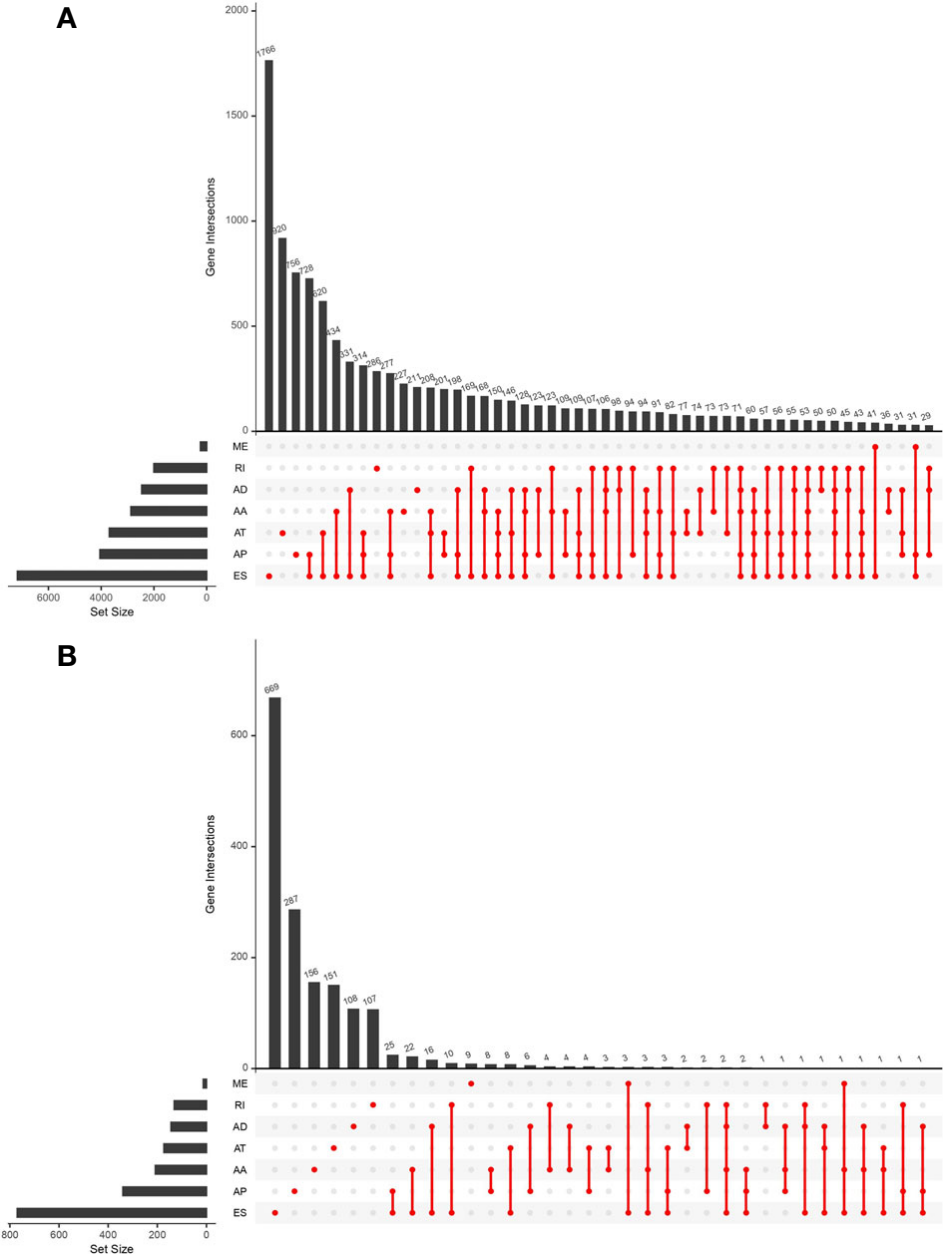
UpSet plot showing the interactions between all alternative splicing (AS) events and parental genes **(A)** and the interactions between AS events associated with survival and corresponding parental genes **(B)** in esophageal carcinoma (EC) patients. Usually, several AS events occurred in a single gene.

This study included 183 patients with EC available in the TCGA database. **Table 1** summarizes the clinical and pathological characteristics of all cases. In addition, an exhaustive analysis of the AS events was carried out. AS events include seven types, namely, alternate acceptor site (AA), alternate donor site (AD), alternate promoter (AP), alternate terminator (AT), mutually exclusive exons (ME), retained intron (RI), and exon skip (ES). The UpSet plot displayed the intersection between each AS event type and its parental genes (**Figure 1A**). The results indicated that a unique gene could have several AS event types, with ES being the main type in EC, while ME was rare.

**Table 1:** Clinicopathological characteristics of 183 patients with esophageal carcinoma from the TCGA database.

Clinicopathological characteristics	Value (%)
Gender, *n* (%)	
Female	27 (14.8)
Male	156 (85.2)
TCGA stage, *n* (%)	
Stage I	18 (9.8)
Stage II	78 (42.6)
Stage III	55 (30.1)
Stage IV	9 (4.9)
Unknown	23 (12.6)
T stage, *n* (%)	
T1	32 (17.5)
T2	43 (23.5)
T3	86 (47.0)
T4	5 (2.7)
Unknown	17 (9.3)
N stage, *n* (%)	
N0	76 (41.5)
N1	68 (37.2)
N2	12 (6.6)
N3	10 (5.4)
Unknown	17 (9.3)
Survival, *n* (%)	
Yes	109 (59.6)
No	74 (40.4)

### Survival-Related AS Events

**Figure 2:**
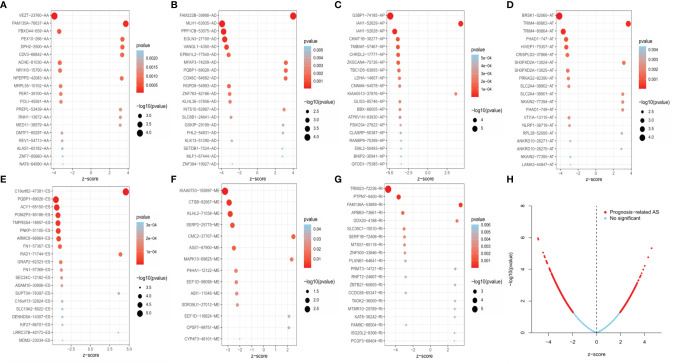
The 20 most relevant prognostic AS events in different types of AS events, except for ME, which had only 14 survival-related AS events **(A–G)**. Volcano plots showed the AS events with (red color) and without (blue color) significant relation with the overall survival (OS) of EC patients **(H)**.

Univariate Cox regression analysis was executed to measure the survival period associated with AS events. A total of 2,212 AS events from 1,623 parental genes were found associated with OS (*P <* 0.05). Therefore, two or more AS events that were significantly related to OS might occur in a single gene. Among the AS event types, ES was the most frequent event related to survival. The UpSet plot revealed the intersection of parent genes and each survival-related AS event type (**Figure 1B**). The 20 most relevant survival-related AS events of each type are shown in **Figures 2A–G**. The distribution of AS events with and without significant relation with OS is shown in **Figure 2H**.

### Construction and Assessment of Prognostic Signatures

**Figure 3:**
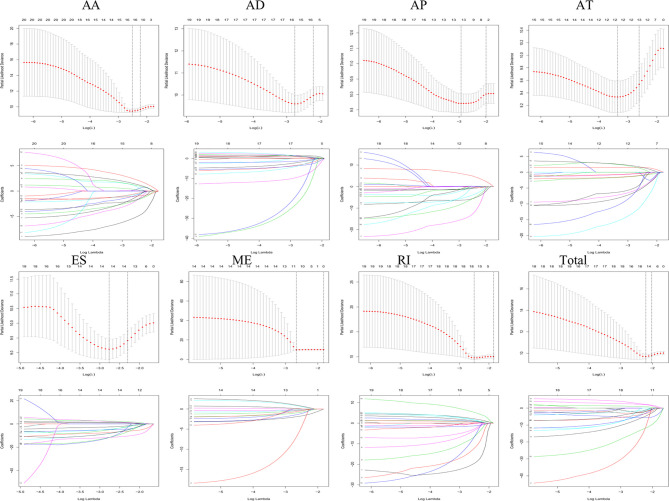
The selection of survival-associated AS events by Lasso regression analysis that avoided overfitting and excluded co-expression.

**Figure 4:**
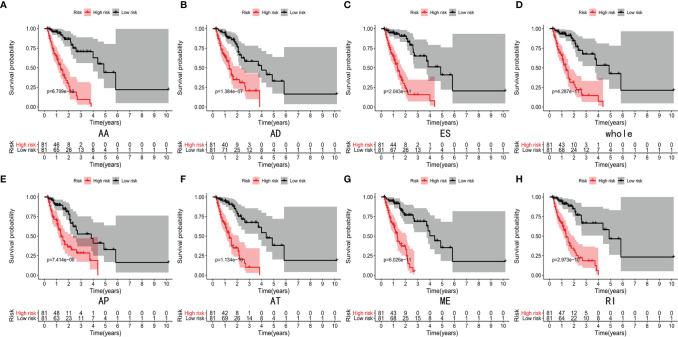
K-M curves analyses showed significant differences of AA type **(A)**, AD type **(B)**, ES type **(C)**, whole type **(D)**, AP type **(E)**, AT type **(F)**, ME type **(G)** and RI type **(H)** in survival time between the low-risk (81 cases) and high-risk (81 cases) groups of EC patients.

**Figure 5:**
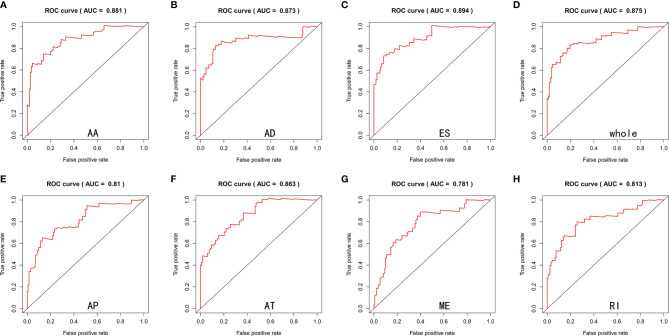
ROC curves estimated the predictive power of AA **(A)**, AD **(B)**, ES **(C)**, whole **(D)**, AP **(E)**, AT **(F)**, ME **(G)** and RI **(H)** type of prognostic signatures.

**Figure 6:**
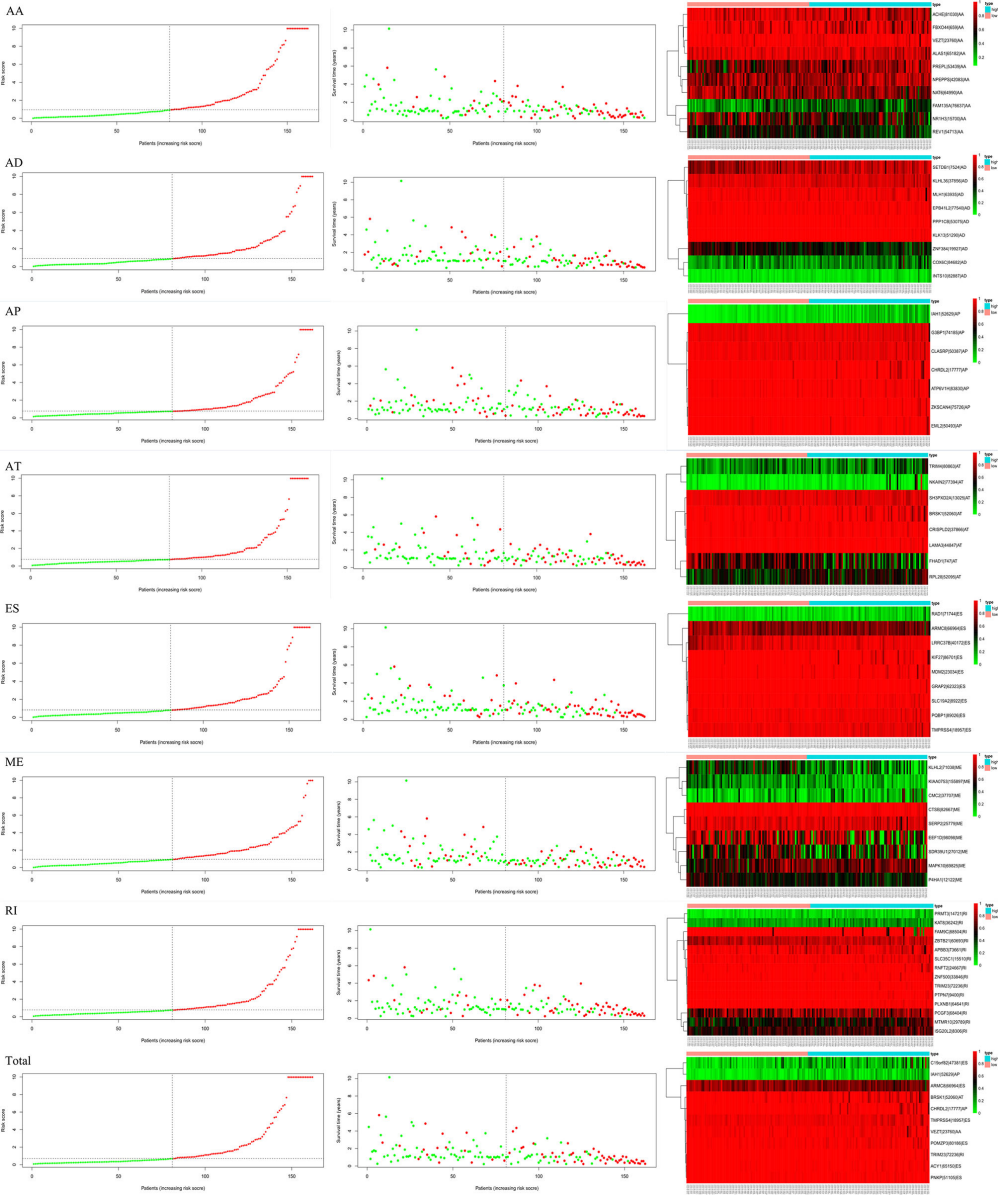
Survival time, risk score distribution, and expression heatmap of AS events associated with survival.

Lasso and multivariate Cox regression analyses were performed to construct the prognostic signatures based on AS events associated with survival. **Figure 3** showed the Lasso regression results that avoided overfitting and excluded AS events with a strong correlation. The most significant survival-associated AS events were selected using multivariate Cox regression analysis. Then, the prognostic signatures were constructed based on seven AS event types and the total of AS events combined. Details of specific AS events in the eight prognostic models are shown in **Table 2**. In addition, EC patients were divided into low- and high-risk groups, using the average risk score as cutoff. The K-M curves revealed that survival outcome differed significantly between the two groups (**Figure 4**). Meanwhile, ROC curves were used to assess the predictive ability of each prognostic signature. The results indicated that ES risk score had the greatest prognosis prediction power, with AUC of 0.894, followed by AA with AUC of 0.881, and AD with AUC of 0.873 (**Figure 5**). The AUCs of the seven prognostic signatures were greater than 0.8. According to risk score distribution, detailed information regarding the corresponding splicing pattern, life status, and survival time of the candidate AS events was shown (**Figure 6**).

**Table 2:** Multivariate Cox analysis of prognostic alternative splicing predicting overall survival.

Type	Gene symbol	Splice-seq CD	AS type	Coef	HR	HR95L	HR95H	*P*-value
AA	*VEZT*	23,760	AA	−8.977418898	0.000126228	3.69E-07	0.043124009	0.002560106
	*FAM135A*	76,637	AA	5.041084173	154.6375781	12.2966322	1944.660959	9.52E-05
	*FBXO44*	659	AA	−4.271616079	0.013959206	0.001061955	0.183491203	0.001153854
	*ACHE*	81,030	AA	−4.612374331	0.009928217	0.000413829	0.238188686	0.004442978
	*NR1H3*	15,700	AA	−1.808748013	0.163859158	0.032583457	0.824032383	0.02817642
	*NPEPPS*	42,083	AA	3.251016751	25.81657562	1.24159728	536.8049587	0.035752682
	*PREPL*	53,439	AA	3.595508855	36.43423502	3.921411216	338.5142256	0.001569822
	*REV1*	54,713	AA	−2.50702885	0.081510058	0.005899686	1.126142915	0.061304796
	*ALAS1*	65,182	AA	−6.123506353	0.002190761	1.70E-05	0.282126177	0.01349319
	*NAT6*	64,990	AA	−2.413831351	0.089471839	0.008497872	0.942025236	0.044464237
AD	*MLH1*	63,935	AD	−9.29900017	9.15E-05	1.95E-06	0.004302092	2.21E-06
	*PPP1CB*	53,075	AD	−47.84186724	1.67E-21	8.52E-30	3.27E-13	9.05E-07
	*EPB41L2*	77,540	AD	−13.46305003	1.42E-06	9.53E-14	21.23659667	0.110176567
	*COX6C*	84,682	AD	2.245686376	9.446897454	0.932196493	95.73504316	0.057361862
	*KLHL36*	37,856	AD	−10.45603993	2.88E-05	1.57E-08	0.052697774	0.006376113
	*KLK13*	51,290	AD	−58.2391438	5.09E-26	5.90E-37	4.40E-15	5.82E-06
	*SETDB1*	7,524	AD	4.62392316	101.8929915	2.245728743	4623.079148	0.017519662
	*ZNF384*	19,927	AD	−4.750719547	0.008645472	0.000437194	0.170963546	0.001808787
	*INTS10*	82,887	AD	6.20851945	496.9649251	0.622384776	396819.0517	0.068623958
AP	*G3BP1*	74,185	AP	−6.941199474	0.000967109	2.17E-07	4.307732264	0.105388563
	*IAH1*	52,629	AP	9.36649356	11690.05267	93.07287085	1468283.188	0.000145645
	*CHRDL2*	17,777	AP	−7.926012434	0.000361224	3.53E-06	0.036949218	0.000788443
	*ZKSCAN4*	75,726	AP	−21.29940519	5.62E-10	1.11E-14	2.85E-05	0.000116776
	*ATP6V1H*	83,830	AP	−8.527017934	0.000198045	1.33E-08	2.956743819	0.082054278
	*CLASRP*	50,387	AP	−14.75419181	3.91E-07	9.21E-11	0.001661865	0.000537411
	*EML2*	50,493	AP	−9.529288129	7.27E-05	1.87E-09	2.821145408	0.077130364
AT	*BRSK1*	52,060	AT	−10.30947299	3.33E-05	3.76E-07	0.002954543	6.63E-06
	*TRIM4*	80,863	AT	2.240855683	9.40137244	1.275915548	69.27245607	0.027871802
	*FHAD1*	747	AT	−2.272320157	0.103072757	0.030631748	0.34682948	0.000242164
	*CRISPLD2*	37,866	AT	−24.55466589	2.17E-11	2.75E-17	1.71E-05	0.000392855
	*SH3PXD2A*	13,025	AT	−11.14442427	1.45E-05	7.92E-09	0.026385412	0.003629589
	*NKAIN2*	77,394	AT	3.693478362	40.18437996	6.25086952	258.3295632	0.000100081
	*RPL28*	52,095	AT	3.377437623	29.29560847	3.265469128	262.8206369	0.00255192
	*LAMA3*	44,847	AT	−17.93411065	1.63E-08	2.56E-14	0.010334742	0.008522519
ES	*PQBP1*	89,026	ES	−12.93831488	2.40E-06	8.67E-11	0.06667806	0.013184588
	*TMPRSS4*	18,957	ES	−14.02952861	8.07E-07	2.63E-10	0.002482335	0.000617259
	*ARMC8*	66,964	ES	−5.565952335	0.003825935	0.00018956	0.077219957	0.000282895
	*RAD1*	71,744	ES	5.8855313	359.7938758	5.421302958	23878.32484	0.005965413
	*GRAP2*	62,323	ES	−27.55111595	1.08E-12	2.70E-18	4.35E-07	2.85E-05
	*SLC19A2*	8,922	ES	−20.70519997	1.02E-09	7.69E-15	0.000134827	0.000579681
	*KIF27*	86,701	ES	−6.343152139	0.00175875	2.78E-05	0.111454195	0.002731329
	*LRRC37B*	40,172	ES	6.632135044	759.1011562	0.658367502	875247.5831	0.06521805
	*MDM2*	23,034	ES	−13.21134193	1.83E-06	5.62E-09	0.000596169	7.64E-06
RI	*TRIM23*	72,236	RI	−27.24958504	1.46E-12	7.89E-21	0.000271645	0.005027545
	*PTPN7*	9,400	RI	−23.62235252	5.51E-11	4.44E-19	0.006830103	0.012977311
	*APBB3*	73,661	RI	−3.459593373	0.031442545	0.000903587	1.094120607	0.056095181
	*SLC35C1*	15,510	RI	−13.49900317	1.37E-06	1.88E-10	0.010011925	0.002935408
	*ZNF500*	33,846	RI	−17.56509623	2.35E-08	4.71E-14	0.011758429	0.008699987
	*PLXNB1*	64,641	RI	−23.25232663	7.97E-11	9.16E-22	6.937108663	0.070411301
	*PRMT3*	14,721	RI	4.401069058	81.53799092	0.418704068	15878.6228	0.101780004
	*RNFT2*	24,667	RI	−8.617420764	0.000180926	1.74E-07	0.187967393	0.015031693
	*ZBTB21*	60,693	RI	4.833594835	125.6618835	0.928677618	17003.64976	0.053555705
	*MTMR10*	29,789	RI	2.36616114	10.65640522	1.157851457	98.07732373	0.036672998
	*KAT8*	36,242	RI	5.667882178	289.4209429	2.478030396	33802.84695	0.019617412
	*FAM9C*	88,504	RI	−1.535596952	0.215327113	0.046258254	1.002324169	0.050346829
	*ISG20L2*	8,306	RI	3.435672729	31.05229533	1.187669134	811.8801927	0.03908868
	*PCGF3*	68,404	RI	1.699826542	5.472997975	0.584050555	51.28615425	0.136508444
ME	*KIAA0753*	155,897	ME	−3.525315753	0.029442509	0.002217588	0.390902827	0.007543125
	*CTSB*	82,667	ME	−20.32439387	1.49E-09	6.70E-15	0.00033158	0.001215278
	*KLHL2*	71,038	ME	−2.172908742	0.113845986	0.030949924	0.418770293	0.001076274
	*SERP2*	25,779	ME	−2.694460892	0.067578804	0.001957137	2.333456684	0.135946286
	*CMC2*	37,707	ME	2.211708856	9.131307153	1.895116227	43.99770797	0.005837023
	*MAPK10*	69,825	ME	2.954969149	19.20113033	2.520599882	146.26812	0.004339624
	*P4HA1*	12,122	ME	−2.444845163	0.086739564	0.004574927	1.644562372	0.103401542
	*EEF1D*	98,098	ME	−1.270932026	0.280570001	0.121598752	0.647371165	0.002889094
	*SDR39U1*	27,012	ME	−0.738332198	0.477910311	0.172952733	1.320581995	0.154519349
Whole	*TRIM23*	72,236	RI	−45.39503761	1.93E-20	6.42E-29	5.79E-12	5.17E-06
	*C19orf82*	47,381	ES	2.302380336	9.997952643	1.813511981	55.11904973	0.008207877
	*ACY1*	65,150	ES	−19.96028565	2.14E-09	1.24E-18	3.702944869	0.065866873
	*IAH1*	52,629	AP	5.239860379	188.6437619	1.56143424	22790.88544	0.032182705
	*POMZP3*	80,186	ES	−13.68308948	1.14E-06	2.21E-11	0.059070021	0.013480575
	*TMPRSS4*	18,957	ES	−8.830874925	0.00014615	5.10E-08	0.418814597	0.029686598
	*PNKP*	51,105	ES	−26.12505519	4.51E-12	2.27E-24	8.942433332	0.070556568
	*BRSK1*	52,060	AT	−10.18431385	3.78E-05	2.86E-07	0.004978673	4.33E-05
	*ARMC8*	66,964	ES	−3.940631323	0.019435941	0.000802912	0.470481911	0.015362605
	*VEZT*	23,760	AA	−8.509043331	0.000201637	4.99E-07	0.081425304	0.005450663
	*CHRDL2*	17,777	AP	−8.171065309	0.000282717	1.64E-06	0.048676876	0.001867045
	*TRIM23*	72,236	RI	−45.39503761	1.93E-20	6.42E-29	5.79E-12	5.17E-06

AA, alternate acceptor site; AD, alternate donor site; AP, alternate promoter; AT, alternate terminator; ES, exon skip; RI, retained intron; ME, mutually exclusive exons.

### Stratified Survival Analysis With Prognostic Characteristics

**Figure 7:**
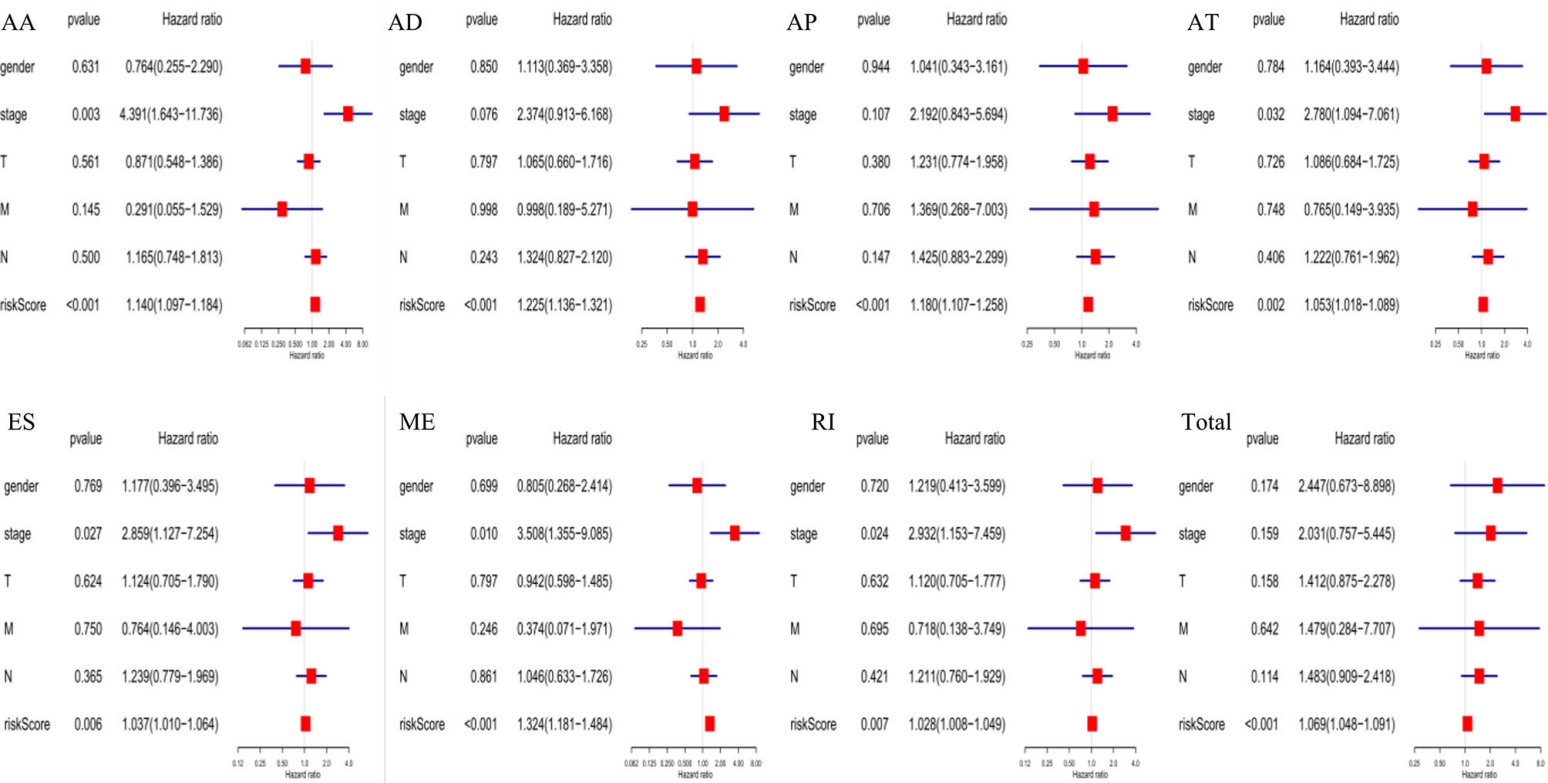
Independent prognostic analysis indicated that the risk score of constructed prognosis signatures could be employed as an independent predictor of EC patients.

The following clinical variables were added to the univariate/multivariate Cox regression analyses: gender; T, M, and N stages; and clinical stage. The results indicated that several clinical characteristics, including high clinical stage, N stage, and high-risk scores, could predict poor survival of EC patients. However, the risk score of prognostic signatures was an independent prognostic indicator (**Figure 7**).

### Functional Enrichment Analyses of SF Genes Associated With AS Events

**Figure 8:**
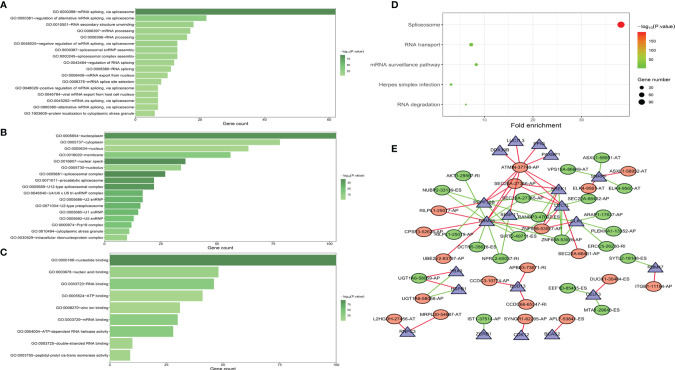
GO functional enrichment **(A–C)** and KEGG pathway enrichment **(D)** analyses of splicing factor (SF) genes that were correlated with AS events associated with survival. The interaction network indicated the hub SFs and AS events **(E)**.

Functional enrichment analyses were performed to reveal the underlying mechanisms of SFs correlated with survival-associated AS events. The BP terms of these genes were mainly associated with “mRNA splicing *via* spliceosome” and “RNA secondary structure unfolding” (**Figure 8A**). “Nucleoplasm,” “cytoplasm,” and “nucleus” were the three most important CC terms (**Figure 8B**). Regarding MFs, “nucleotide binding,” “nucleic acid binding,” and “RNA binding” were the most abundant categories (**Figure 8C**). KEGG analysis demonstrated five significantly enriched pathways, namely, “spliceosome,” “mRNA surveillance pathway,” “RNA transport,” “Herpes simplex infection,” and “RNA degradation” (**Figure 8D**).

### Construction of AS Events–SF Genes Correlation Network

A correlation network was established between SF genes and AS events associated with survival. In total, 19 downregulated AS events, 21 upregulated AS events, and 20 SFs were found in the network (**Figure 8E**). The five most important nodes were selected according to their grade, including three AS events (RANBP3-47007-ES, ATMIN-37748-AP, and SEC23A-27346-AP) and two SFs (RBM25 and PRPF38B) (**Table 3**).

**Table 3:** The correlation of hub SF genes and AS events.

SF	AS event	Correlation coefficient	*P*-value	Regulation
PRPF38B	ATMIN-37748-AP	−0.721723263	3.96E-24	Negative
	NUBP2-33139-ES	0.610094073	7.66E-16	Positive
	RANBP3-47007-ES	0.686152021	4.38E-21	Positive
	RILPL1-25077-AP	−0.600478954	2.82E-15	Negative
	RILPL1-25079-AP	0.603260312	1.94E-15	Positive
	SEC23A-27345-AP	0.636096569	1.80E-17	Positive
	SEC23A-27346-AP	−0.636096569	1.80E-17	Negative
	SIRT2-49711-ES	0.637655807	1.42E-17	Positive
RBM25	AKT1-29567-RI	0.608457747	9.59E-16	Positive
	ATMIN-37748-AP	−0.718782166	7.36E-24	Negative
	CPSF3-52626-AP	−0.614632102	4.08E-16	Negative
	DCTN5-35626-ES	0.657157877	6.59E-19	Positive
	NPRL2-65037-RI	0.612639339	5.39E-16	Positive
	NUBP2-33139-ES	0.696776504	6.01E-22	Positive
	RANBP3-47007-ES	0.71012252	4.37E-23	Positive
	RILPL1-25077-AP	−0.645552245	4.21E-18	Negative
	SEC23A-27345-AP	0.636145963	1.79E-17	Positive
	SEC23A-27346-AP	−0.636145963	1.79E-17	Negative
	SIRT2-49711-ES	0.753508244	2.81E-27	Positive
	UBE2V2-83797-AP	−0.602237229	2.23E-15	Negative
	ZNF638-53926-AP	0.64452843	4.94E-18	Positive
	ZNF638-53927-AP	−0.644517677	4.95E-18	Negative
DDX39B	ATMIN-37748-AP	−0.624264455	1.04E-16	Negative
LUC7L		−0.625868699	8.22E-17	Negative
LUC7L3		−0.602587404	2.12E-15	Negative
PAXBP1		−0.600521622	2.80E-15	Negative
PPIG		−0.60482944	1.57E-15	Negative
PRPF38B		−0.721723263	3.96E-24	Negative
RBM25		−0.718782166	7.36E-24	Negative
SREK1		−0.658072096	5.67E-19	Negative
SRSF11		−0.62348331	1.16E-16	Negative
CLK1	RANBP3-47007-ES	0.602939294	2.03E-15	Positive
PRPF38B		0.686152021	4.38E-21	Positive
RBM25		0.71012252	4.37E-23	Positive
SREK1		0.638355082	1.28E-17	Positive
PRPF38B	SEC23A-27345-AP	0.636096569	1.80E-17	Positive
RBM25		0.636145963	1.79E-17	Positive
LUC7L		−0.629841693	4.59E-17	Negative
PRPF38B		−0.636096569	1.80E-17	Negative
RBM25		−0.636145963	1.79E-17	Negative
SREK1		−0.612415838	5.56E-16	Negative

**Figure 9:**
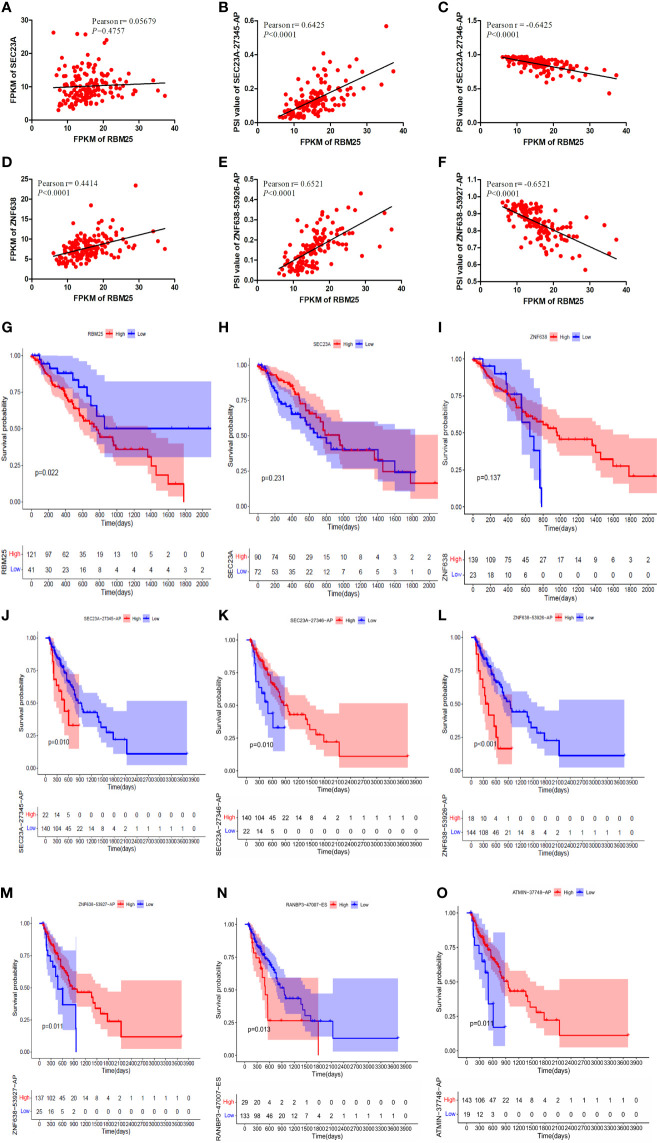
The correlation analysis was performed using Pearson *t*-test and K-M curves. **(A)** There was no correlation between the expression of RBM25 and SEC23A (*P* = 0.4757). **(D)** There was a weak correlation between RBM25 and ZNF638 (*P <* 0.0001). RBM25 could positively regulate SEC23A-27345-AP **(B)** (*P <* 0.0001) and ZNF638-53926-AP **(E)** (*P <* 0.0001) and negatively regulate SEC23A-27346-AP **(C)** (*P <* 0.0001) and ZNF638-53927-AP **(F)** (*P <* 0.0001). RBM25 **(G)** (*P* = 0.022), SEC23A-27345-AP **(J)** (*P* = 0.010), and ZNF638-53926-AP **(L)** (*P <* 0.0001) were all negatively related to the OS of EC patients. On the contrary, SEC23A-27346-AP **(K)** (*P* = 0.010) and ZNF638-53927-AP **(M)** (*P* = 0.011) were positively related to the OS consistent with the regulation of expression level. The parental genes *SEC24A* **(H)** (*P* = 0.231) and *ZNF638* **(I)** (*P* = 0.137) were not the prognosis predictors of EC. RANBP3-47007-ES **(N)** was negatively related to the OS of EC patients. The patients with low PSI of RANBP3-47007-ES had better outcomes (*P* = 0.013). ATMIN-37748-AP **(O)** and SEC23A-27346-AP **(I)** were positively related to the OS of EC patients. The patients with high PSI of ATMIN-37748-AP had better outcomes (*P* = 0.011).

Our results demonstrated that RBM25 could positively regulate eight AS events and negatively regulate six AS events. Interestingly, we found that RBM25 could positively regulate SEC23A-27345-AP and ZNF638-53926-AP and negatively regulate SEC23A-27346-AP and ZNF638-53927-AP. **Figures 9A, D** indicate that there were no correlation between the expression of RBM25 and SEC23A (*r* = 0.05679, *P* = 0.4757) and a weak correlation between RBM25 and ZNF638 (*r* = 0.4414, *P <* 0.0001). A total of 139 EC tumor tissues were used to show the correlation between the expression of RBM25 and PSI value of SEC23A-27345-AP (**Figure 9B**, *r* = 0.6425, *P <* 0.0001), SEC23A-27346-AP (**Figure 9C**, *r* = *−*0.6425, *P <* 0.0001), ZNF638-53926-AP (**Figure 9E**, *r* = 0.6521, *P <* 0.0001), and ZNF638-53927-AP (**Figure 9F**, *r* = *−*0.6521, *P <* 0.0001). Furthermore, we analyzed the correlation between RBM25 and AS events, with parental genes as prognosis markers. Our results demonstrated that RBM25 (**Figure 9G**), SEC23A-27345-AP (**Figure 9J**), and ZNF638-53926-AP (**Figure 9L**) were all negatively related to the OS of EC patients. On the contrary, SEC23A-27346-AP (**Figure 9K**) and ZNF638-53927-AP (**Figure 9M**) were positively related to the OS which was consistent with the regulation of expression level. However, the parental genes *SEC24A* (**Figure 9H**) and *ZNF638* (**Figure 9I**) were not the prognosis predictors of EC. Both the expression and prognosis analysis results confirmed that it was more valuable to explore the correlation between SFs and AS events than parental genes.

Meanwhile, the relationship between the other two most important hub AS events (RANBP3-47007-ES and ATMIN-37748-AP) and the prognosis of EC patients were also performed. Our results indicated that RANBP3-47007-ES (**Figure 9N**) was negatively related to the OS which was upregulated in EC patients. On the other hand, patients with low PSI of RANBP3-47007-ES had better outcomes (*P* = 0.013). By contrast, ATMIN-37748-AP (**Figure 9O**) was positively related to the OS which was downregulated in EC patients. Patients with high PSI of ATMIN-37748-AP had better outcomes (*P* = 0.011).

## Discussion

AS event is the mechanism of producing several mRNA variations from a single transcript (25, 26). Most human genes are alternatively spliced to produce RNA isoforms that encode functionally different proteins (27). The AS event is the primary step of gene dysregulation in many diseases and plays an essential role in several biological processes. Growing evidence has shown that AS event participates in tumorigenesis and has prognostic value in cancer patients (28, 29). A strong interaction between AS events and SFs has also been reported by previous studies (6, 22). SFs that regulate AS processes are dysregulated or mutated in various diseases, including cancer (30, 31). Recent studies on several cancer types have shown that abnormal regulation of SFs could reduce cell proliferation, lead to the production of abnormal mature transcripts that drive tumorigenesis, or promote cellular senescence by regulating AS events (13, 28, 29, 32).

To our knowledge, this is the most comprehensive study in which abnormal AS variants and SFs have been explored in EC patients employing high-throughput TCGA data. Here, we studied AS events associated with survival and constructed prognostic signatures in EC patients. Univariate Cox regression analysis revealed 2,212 AS events related to survival outcomes. Multivariate Cox regression analysis was performed after Lasso regression analysis and eight prognostic signatures were constructed based on the seven AS event types and total AS events combined. The EC patients were divided into low- and high-risk groups according to the risk score. K-M analysis revealed a significant difference in the survival of patients between these two groups. In addition, the AUC values of ES, AA, AD, AT, AP, RI, and total events were all greater than 0.8, suggesting that these seven prognostic signatures were valuable in predicting the prognosis of EC patients. Nonetheless, a small number of studies on ES, AA, AD, AT, AP, and RI events in EC patients have been conducted so far. Therefore, further studies should be performed to better understand the effects of these seven AS event types in EC patients.

As SFs are one of the most important regulators of AS events, GO and KEGG enrichment analyses were performed on SFs which were significantly related to AS events to clarify their potential mechanisms in EC patients. The results showed that “mRNA splicing *via* spliceosome” and “RNA secondary structure unwinding” were the two most important BP and “nucleoplasm,” “cytoplasm,” and “nucleus” were the three most important CC terms. Regarding MFs, “nucleotide binding,” “nucleic acid binding,” and “RNA binding” were the three most abundant categories. GO analysis showed that these SFs were significantly related to splicing. KEGG results indicated that the spliceosome was the most significantly enriched pathway. In addition, we have also built an interactive network between survival-related AS events and SFs. The five most important nodes as hub SFs or hub AS events were selected in accordance with their grade. Among the selected hub SFs and AS events, RANBP3-47007-ES was upregulated, ATMIN-37748-AP and SEC23A-27346-AP were downregulated, and two SFs (RBM25 and PRPF38B) were dysregulated. RBM25 is a global splicing factor that can promote the inclusion of other splicing exons, and is regulated by lysine monomethylation (33). RBM25 is also reported as a new type of tumor suppressor that can control the splicing of key genes (34). Our results demonstrated that RBM25 could regulate 14 AS events in the interactive network. Interestingly, we found that RBM25 could positively or negatively regulate two different AS events of SEC23A and ZNF638. A total of 139 EC tumor tissues were used to show the positive correlation between the expression of RBM25 and PSI value of SEC23A-27345-AP and ZNF638-53926-AP and the negative correlation between RBM25 and PSI value of SEC23A-27346-AP and ZNF638-53927-AP. Meanwhile, our results indicated that there were no correlation between the expression of RBM25 and SEC23A and a weak correlation between RBM25 and ZNF638. Furthermore, our results demonstrated that RBM25, SEC23A-27345-AP, and ZNF638-53926-AP were all negatively related with the OS of EC patients. On the contrary, SEC23A-27346-AP and ZNF638-53927-AP were positively related to the OS which was consistent with the regulation of expression level. The parental genes *SEC24A* and *ZNF638* were not the prognosis predictors. Actually, *SEC23A* and *ZNF638* genes have eight and nine different AS events, respectively, showing different expression levels in EC tissue. Therefore, it is more valuable to analyze the correlation between SFs and specific AS events, comparing with parental genes. The regulation network of SF genes and AS events can better explore the potential mechanism of tumorigenesis and progression. ATMIN has been demonstrated to be a tumor-suppressor gene in lung adenocarcinoma (35) and an essential developmental transcription factor (36). The loss of ATMIN can cause chromosome segregation defects (37). The role of ATMIN in EC is much less clear, and whether ATMIN and RBM25 function in synergy in EC is unknown and needs further verification.

There are certain limitations in our study that deserve to be addressed. Firstly, since there was no additional external cohort for splicing data, we only used data from the TCGA database to evaluate the survival-related AS events of EC patients without verification. Secondly, the number of patients in this retrospective study was limited. Therefore, studies with larger sample sizes are necessary to validate the results obtained here. Finally, the interaction between hub SFs and AS events needs to be validated with clinical samples. Also, it is of great significance to study the mechanism of these genes in EC.

## Conclusion

This is the first study to comprehensively investigate survival-related alternative splice variants and construct prognostic signatures in EC patients. The network of interactions between survival-related AS events and SFs might provide new insights into the underlying mechanism of EC development. In addition, the role of hub AS events and SFs in tumorigenesis and progression of EC needs further studies to be fully clarified.

## Data Availability Statement

Clinical and transcriptome data from esophageal cancer patients were acquired from the TCGA (The Cancer Genome Atlas) database (https://portal.gdc.cancer.gov/). The PSI (Percent Spliced In) values for AS events were downloaded from the TCGA Splice-seq (https://bioinformatics.mdanderson.org/TCGASpliceSeq/), which is a collection of alternative mRNA splicing in cancer.

## Author Contributions

PY, YY, LZ, and GZ contributed to the conception and design of the study. PY wrote the original draft. YY organized the database. GZ performed the statistical analysis. LZ wrote sections of the manuscript. All authors contributed to the article and approved the submitted version.

## Funding

This work was supported by a grant from the Key Research and Development Project of Shandong Province (2019GSF108304).

## Conflict of Interest

The authors declare that the research was conducted in the absence of any commercial or financial relationships that could be construed as a potential conflict of interest.

## Publisher’s Note

All claims expressed in this article are solely those of the authors and do not necessarily represent those of their affiliated organizations, or those of the publisher, the editors and the reviewers. Any product that may be evaluated in this article, or claim that may be made by its manufacturer, is not guaranteed or endorsed by the publisher.
